# Encountering pain: images as a tool for collaborative approaches to pain medicine

**DOI:** 10.1097/j.pain.0000000000003973

**Published:** 2026-04-15

**Authors:** Deborah Padfield, Joanna M. Zakrzewska

**Affiliations:** a Slade School of Fine Art, University College London, London, United Kingdom; b City St George's, University of London, London, United Kingdom; c Royal National ENT & Eastman Dental Hospitals, Euston, London, United Kingdom,; d Pain Management Centre, National Hospital for Neurology & Neurosurgery, UCLH NHS Foundation Trust, London, United Kingdom.

**Keywords:** PAIN, Art, Photography, Medical and healthcare humanities, Arts and health, Transdisciplinary, Communication

## Abstract

Chronic pain remains a multifactorial complex common condition, difficult for patients to communicate or fully understand. We argue the arts can raise public awareness, play a role in capturing patients' subjective narratives, improve wellbeing, and increase mutual understanding in the clinic. Through sharing findings from over 2 decades evidencing the value of photographic images as both assessment and communication tools, we demonstrate images can facilitate democratic and collaborative interaction, enabling patients to retain ownership of their illness experience. We describe how the cocreation of photographic images by a visual artist with chronic pain patients enabled individual pain meanings to emerge. The images were subsequently tested in NHS clinics with patients not involved in making them, and their impact evaluated from a variety of perspectives: linguistic; nonverbal, clinical, and psychotherapeutic. Through a series of projects, it was discovered that the visual language did not necessarily *replace* verbal language but *reinvigorated* it. Patients used the images to talk about their emotional and subjective states, the impact pain had on their lives and voice issues that mattered most to them, aligning with patient-centred care. Increased rapport and clinician patient affiliation was observed with image use and the value of an active shared physical space between clinician and patient. We posit that eliciting pain meanings is as valuable as measurement in a chronic pain context and that images can significantly increase validation. Cocreating an international set of images/pain cards could provide an economic and fruitful starting point for pain conversations and assessment globally.

## 1. Introduction

**Figure 1.**
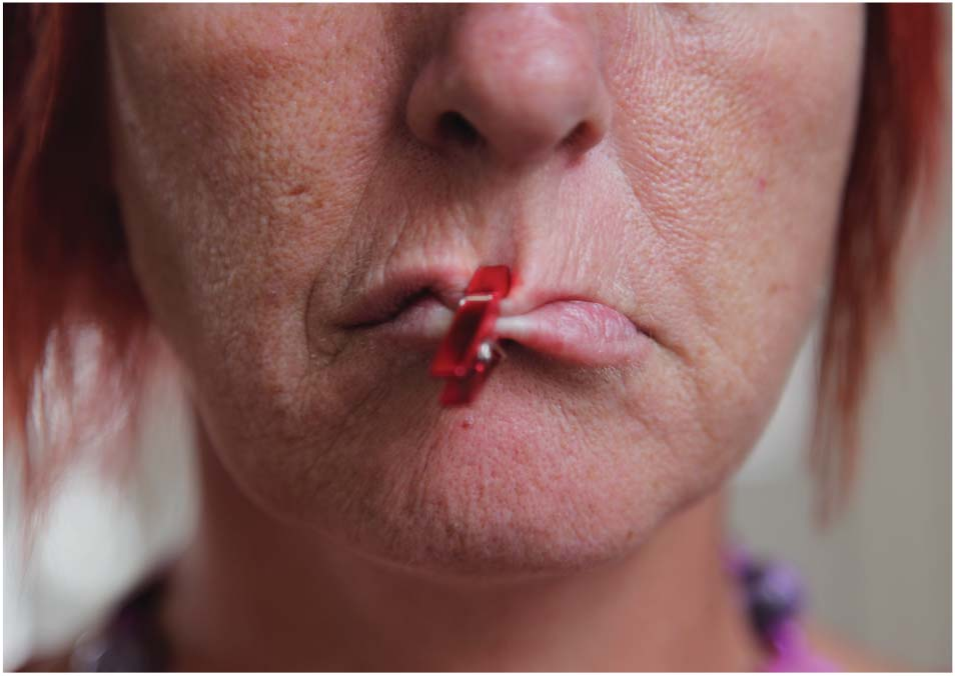
Deborah Padfield with Liz Aldous, “Untitled” from the series Face2face, 2008–13. Digital Archival Prints. © Deborah Padfield.

Pain is more than a collection of symptoms, it holds meaning. For clinicians and patients alike, that there is no “cure” or easy answer to chronic pain is a huge challenge. We suggest images can offer another means of enhancing the process of eliciting and unpicking meaning most likely to benefit patients. Despite most diagnoses of chronic pain coming through history taking, doctors are not trained in meaning, in the way those in the humanities are.^102^ Such training might help them interpret patients' gestures and pauses, understand the symbolism buried in their language, and interrogate the nature of healing and “what it means” to diverse individuals and communities. Attunement to the meaning of a patient's experience will not only improve doctor–patient communication but can also have demonstrable effects on the pain level itself or on the patient's capacity to live despite it. Our work over the past 2 decades has revealed ways in which images and image-making processes can open up discussion around meaning; support face-to-face discussion between clinicians, carers, and people living with pain; and effect collaborative dialogue between those living with and those witnessing pain. These can be valuable tools for the future, both within healthcare practice and, although not the focus of this article, by extension, public and medical education (Fig. 1).

In an age where technology is significantly relied on and frequent use of phone or video consultation, a return to face-to-face interaction, to collaborative dialogue and reminders of the physicality rather than abstract or theoretical nature of chronic pain, becomes increasingly valuable. The visual arts, and in particular photography, can offer direct ways of eliciting those personal metaphors and meanings and approaching the paradoxes of chronic pain. There is validation for a patient in having their story seen, heard, and believed^6,31^ and the converse negative impact of invalidation well-recognised.^100^ As evidenced by the wealth of photoelicitation, photovoice,^14,23,28,37,97^ and phototherapy projects,^26,53^ photographic images can be a way of validating pain stories and visualising and drawing out narratives into a sharable visible space. Pioneer of narrative medicine, Rita Charon, has convincingly argued that sickness and healing can be understood as narrative acts.^18,19^ Psychometricians and study methodologists have subsequently examined the outcomes of narrative training in health professions settings in systematic reviews and scoping reviews,^38,41,59^ including specialty practices dealing with pain control in particular.^74^ Clinical and translational science journals and general medical journals have published outcome data and theoretical frameworks that support the conceptual constructs of photographic interventions in pain medicine.^20,77^ The substantiating evidence for the case for narrative contributions to patient outcomes emerges from philosophical and aesthetic literature as well as clinical and basic science literature because the conceptual investigations are just now being formulated.^21^

Scarry has famously asserted “to have pain is to have certainty; to hear about pain is to have doubt”^81^ (p 13). Images, as our studies evidence,^4^ help return autonomy to the “teller,” eliciting narratives of pain and bringing legitimacy to them in the consulting space, through physically, visibly, and tangibly providing a shared reference point through which to navigate competing understandings. These are so often a stumbling block to effective communication in pain consultations.^42^ The process of moving between shared image and verbal language facilitates patient-centred management, as “the images bring the physicality of pain sensation directly into the room through the materiality of the image on the one hand, and symbolically as a springboard for discussion of the emotional and social impacts of pain on the other”^66^ p. 148.

Chronic pain places a heavy burden on individuals and financial burdens on healthcare systems.^47^ It does not lend itself easily to a biomedical model and therefore alternative means of communication need to be found. “Doctors are not listening, and it is making us unwell.”^27^ Dhairyawal et al. emphasise the value of doctors collaborating and listening, proposing that diseases, such as chronic pain, that are not easily diagnosed or treated and which rely on patient testimony are often less valued. They attract less research funding, leading to less accumulated knowledge and to marginalisation of “undeserving” groups of people.^27^ Images are an inexpensive and effective way of addressing this and eliciting nuanced patient narratives which can add to the knowledge base around this debilitating condition and reduce marginalisation. Bourke^10^ asserts that how we envisage and understand pain directly affects how we experience it. “*People perceive pain as through the prism of the entirety of their lived experiences, including their sensual physiologies, emotional states, cognitive beliefs, and relational standing in various communities”* (p. 13). Pain is thus shaped by the experiences and contexts it develops within. It is therefore urgent we explore the meanings and symbolisms pain generates and how these are deep-rooted in sociocultural contexts. Images are an ideal method for exploring and revealing these meanings and symbolisms as they tap into our unconscious emotions and individual and collective values and beliefs. Throwing the consultation dialogue off its standard track, disrupting the habitual pattern of listener and speaker where the clinician does most of the speaking^61,87^ provides an opportunity for patients to take control of the narratives around their pain^84^ (pp. 271-2). Creativity and imagination, involved in both the creating and viewing of the images, draw on the less conscious part of ourselves, bringing forth observations and feelings which surprise us.^94,95^ The process of negotiating between images and the words they generate, thus enables participants to see what they might not expect to see and to hear what they might not expect to hear—arguably the basis of fruitful dialogue. It is the ability of photographic images to initiate such a dialogic process, revealing new meanings potentially allowing recognition of as yet unarticulated feelings and associations that this article advocates for.

## 2. Meanings as important as measures

Patient narratives, although socially constructed, are an essential part of diagnosis and management. With limited verbal language available to describe pain^29,81^ and many metaphors overused and focusing on notions of injury^8,9^ or the dysfunctional body,^10^ it is hard to cross the divide between the person living with and the person witnessing/treating pain. It is challenging to unearth meanings which are hidden deep in the body and the psyche, “*Through their life experiences, individuals learn the concept of pain.”*^80^ This is exacerbated in contexts where verbal language or cultural pain conceptions of clinician and patient are different, and/or where there are competing agendas and imperatives “… the interpretation of pain behaviours is heavily dependent on the social and cultural learnings and understandings of both the person in pain and the observer.”^98^ Our work over two decades (along with that of others in this growing field) explores and evidences ways in which images can support assessment and communication, reimagining and opening up spaces where patients can lead the exploration and interpretation of their experience in the safe presence of an informed ‘other.’ Through such a collaborative dialogic process, they can discover a way forward while retaining ownership of their illness experience. We identify our main findings from this process and recommend areas for future research and work.

## 3. The value of the arts to health care and pain medicine

With the change in demographics, socioeconomic contexts driving pain and ever stretched health budgets, we believe it is essential and cost-effective to engage the arts and humanities to improve our approach to chronic pain in the future. The medical humanities is a highly interdisciplinary, “rapidly expanding and increasingly globalised” field^99^ (p. 1). Although constantly evolving, it has long examined “the relationship between health and society, situating medicine and disease within their political, social, historical, ethical, and cultural contexts. It often uses ideas, tools, and methods from disciplines such as history, art, philosophy, theology, and literature to create innovative strategies for understanding and improving health and health care.”^63^ In its early stages or “primal scene,” as described by Whitehead and Woods, it “placed a humanist emphasis on individual protagonists and the role of narrative, metaphor, and gaps in communication within the dynamics of the clinical interaction”^99^ (p. 2). Rather than addressing what could be seen as a simplistic art/science divide, the more recent term of “healthcare humanities,” increasingly including creative as well as clinical practitioners and humanities academics within its fold, describes the current model of the “critical medical humanities” as espousing the principle of “entanglement.” Entanglement proposes moving away from binary distinctions to a notion of all the disciplines enriching each other. This latest “wave” thus proposes a more complex understanding of the relationship between medical/health care/arts and humanities thinking,^34^ which is seen as positive.

The arts and humanities are increasingly recognised as able to play a key role in health promotion with improved recognition of their value and growing rates of social prescribing of arts activities.^22^ Social prescribing is the referral by healthcare professionals to sources of support within the community, including art workshops.^12,13,22^ In the United Kingdom, there is a dedicated “social prescribing day” in the calendar, albeit caution coming from some voices.^92^ Fancourt et al.'s work on the WHO Arts and Health evidence synthesis report^32^ rigorously evidences the many ways in which “Including the arts in health care delivery has been shown to support positive clinical outcomes for patients while also supporting other stakeholders, including healthcare providers, patients' loved ones, and the wider community.” Benefits of engagement with arts activities are seen across several markers, including health promotion and the management of health conditions and illness.^5,32,33,39,46,56,75^ The National Centre for Creative Health (NCCH) was set up in 2021 in the United Kingdom, stemming from a recommendation in the All-Party Parliamentary Group on Arts Health and Wellbeing report “Creative Health,”^1^ which called for a strategic centre for creative health at the UK national level to foster the conditions for creative health to be integral to health and social care and wider systems. Their 2023 Review advocates that creative health is fundamental to a healthy and prosperous society, and its benefits should be available and accessible to all.^2^ Along with other UK organisations such as London Arts & Health, the Culture, Health, and Wellbeing Alliance and others worldwide such as the engagement programme of the National Academies of Sciences, Engineering, and Medicine and The International Arts and Mind Lab Centre for Applied Neuroasthethetics (IAM Lab) at John Hopkins School of Medicine in the US,^50^ and many university centres and hospital charities, they evidence active engagement with the arts, culture, and creativity is beneficial for health and wellbeing.

It is possible to speculate on the reasons this might be, but distinctive to art is its ability to materially embody emotions, feelings, and thoughts difficult to capture through language alone and although often relevant to the intensity and prolongation of pain, frequently perceived as not sufficiently “medical” to find their way into the clinic. An image could be seen as an object not only able to mediate between conscious and unconscious emotions^16^ but also “through which unconscious transactions maybe acted out”^82^ (p. 127), supporting negotiation of unconscious dynamics between clinician and patient and thus supporting the navigation of the well-documented diverse perspectives, understandings, and agendas which can exist at the beginning of a pain consultation.^42^

## 4. Photography and medicine

Photography could be seen as a particularly apposite art medium for documenting and validating subjective experiences of chronic pain. Although we know that photographic images are frequently manipulated, or even AI-generated, we still ascribe to them an ability to document experience we have not personally witnessed—they act as evidence.^7^ As renowned photographic theorist Susan Sontag noted “fake painting … falsifies the history of art” whereas “a fake photograph … falsifies reality”^88^ (p. 86). It is the very distance between the original event and the act of viewing and the distance between the original “intention” and “effect”^88^ (p. 78-9) that allows the photographic image to reveal and validate simultaneously while providing a safe “distance” enabling discussion. Photography becomes both “a way of making known and shaping experience^79^ (p. 270)—both how it is perceived and recorded.

When we started our work visualising pain photographically, there were very few projects exploring images as a means of pain assessment or even as a way for patients to articulate their experience. A handful of projects attempted to expand the “pain drawing” as a means of assessment. As far back as 1988, Uden, Astom, and Bergenudd^96^ argued that drawings might speed up differential diagnosis. There have been subsequent projects which tried to quantify drawings or pictograms, or use them to achieve differential diagnosis.^45,56,57,89,90^ However, these provided patients with little opportunity to explore the meaning of pain in their terms, rather than in a medical framework. As their authors acknowledge, they sometimes revealed the limitations of quantitative approaches to the use of images. The unique value of images to pain medicine lies not necessarily in their ability to quantify pain or aid differential diagnosis, but perhaps in their ability to connect to the unconscious, to reveal to patients aspects of their pain not previously recognised or articulated, and provide shared visual reference points expanding conversation around pain in the clinic. It is through an opening up of dialogue rather than diagnosis, that images come into their own. There have been projects which explore the expressive quality of patients' drawings,^48,60,76^ whose analyses conclude that “Pain-related images can stimulate reflection allowing the inexpressible to be expressed^60^ and provide valuable insights into people's pain worlds, with images reflecting pain cognitions and barriers to recovery. Clinicians may find drawing a helpful tool in the assessment and management of chronic pain, enabling a visual and shareable language for pain.”^76^ An effective way to capture the richness and complexity of an individual's experience may be through fine art, its materiality, polysemy, and in particular, photography with its concomitant association with documentation.

Supported by the Wellcome Trust, artist Alexa Wright explored phantom limb pain with neuropsychologist Peter Halligan, neurologist John Kew, and 8 people with amputated limbs, creating a powerful series of photographic images *After Image*, which effectively made visible the invisible experience of phantom limb pain. The series was shown at the Wellcome Trust in 1997 and subsequently toured in London and globally. Wright had also begun collaborating with Charles Pither et al on exploring pain more widely to include the pain of extreme physical effort such as climbing and physical endurance, *Feeling Hurt* (2001) and *PUSH* (2003). Our work, specifically with patients living with chronic pain and subsequent collaborations with academics and clinicians was, as far as we know, the first to explore photographic images as clinical communication tools and evidenced the value of images to pain assessment, expanding possible dialogue around pain in the clinic.^4,67,69,73^ It raised questions such as: can images act as agents and mediators within clinical interactions around pain; in what ways can creative practice enhance medical practice and vice versa; how can visual language reinvigorate verbal language; in what ways can an art object be viewed as relational; can an image perform a mediating role within the triangle of patient, image, and clinician; and what are the challenges and benefits of a cocreative process to an artist's practice and perhaps to clinical patient interaction?

Rewardingly, it has been generative of further work in the field which continues to expand, including exploration of pain expression through social media images,^36,51,52,60,76^ pain pictograms,^57,91^ and organisations open to the public such as Pain Exhibit,^25^ Exhibiting Pain, the Unmasking Pain Project,^40^ and Chronic Pain Project^24^ to name only a few. In this essay, we give a brief summary of our own initial work, its starting point, subsequent projects and future transcultural ambitions, along with advocating for increased involvement of photography, the arts and humanities in the pain medicine/health care of the future.

## 5. Our starting point—beginnings and discoveries

There are numerous scales in use which aim to capture the wide variety of domains that are important when assessing pain, ranging from simple numeric ones to psychological and linguistic rating scales, including the McGill Pain Questionnaire (MPQ).^58,85^ Patients generally reach for their own metaphors in an attempt to make sense of what is happening to them, usually of injury, but these seldom correlate with the physiological mechanism or the complexity of their subjective experience. Measures such as these struggle to capture the richness and complexity of an individual's experience in the way art can. They were not designed to expand conversation nor aid consultation dynamics through engaging all speakers cognitively and emotionally in the way images can.

In the context of supporting that reach for metaphor and expression, we therefore set out to explore whether we could create an alternative visual language for pain in the hope that images could expand the language and positively affect the interaction which happens around pain in the clinic. Unexpectedly, we discovered through these projects that visual language did not necessarily *replace* verbal language but *reinvigorated* it as patient and clinician went back and forth between the visual image and the language they used to discuss it, evolving more nuanced language and creating in effect a mediating space through which to navigate and unpick meaning.^72^ In her celebrated book, the Story of Pain, Bourke^10^ alludes to the clues contained in metaphors people use for pain. Giving a list of verbal metaphors for pain, she notes that the “more mechanistic metaphors” imply a “detached concern for the dysfunctional body that can be taken to the repair-shop” triggering an expensive process. On the other hand, metaphors arising out of patient narratives, however surreal, encourage accurate diagnosis^10^ (p. 59), mutual understanding, and an awareness of the social, cultural, and biographic elements within that experience. Alternative discourses to dominant frameworks, such as medicalised language, are important to generate through cocreated methodologies, such as cocreated images, able to embody subtle and cultural expressions of pain not necessarily communicated through oral or written language. These can enrich the discourses with vulnerable populations in future work, inform pain services such as the NHS, improve equity of care and effectiveness of pain communication with global impact.

### 5.1. Perceptions of pain (2001-2006) and Face2face (2008-2013)

*Face2face* (2008-2013) was a collaboration with patients and staff from a UK healthcare provider. It built on an earlier collaboration, *Perceptions of Pain* (2001-2006) at another UK healthcare unit with input from a primary care provider. Both projects involved working 1:1 with patients during pain management programmes, to cocreate photographic images with them, led by what they wanted or needed to communicate. The process of cocreating the images has been well-documented,^64–66,70^ but it is worth noting the fact that the creative process was a cocreative one. Both the person living with pain and the artist had input into the creative process, which stemmed from a desire not to reappropriate painful experiences, which can all too easily happen, but to combine and build on both their creative strengths and imaginations while doing justice to the very intense experiences they reflected. The process was a genuinely living process. In some ways, it paralleled unpredictable processes sometimes seen in the consulting room, focusing participants on the here and now of the interaction, rather than rehearsed stories which can have lost their meaning or authenticity through telling and re-telling.

**Figure 2.**
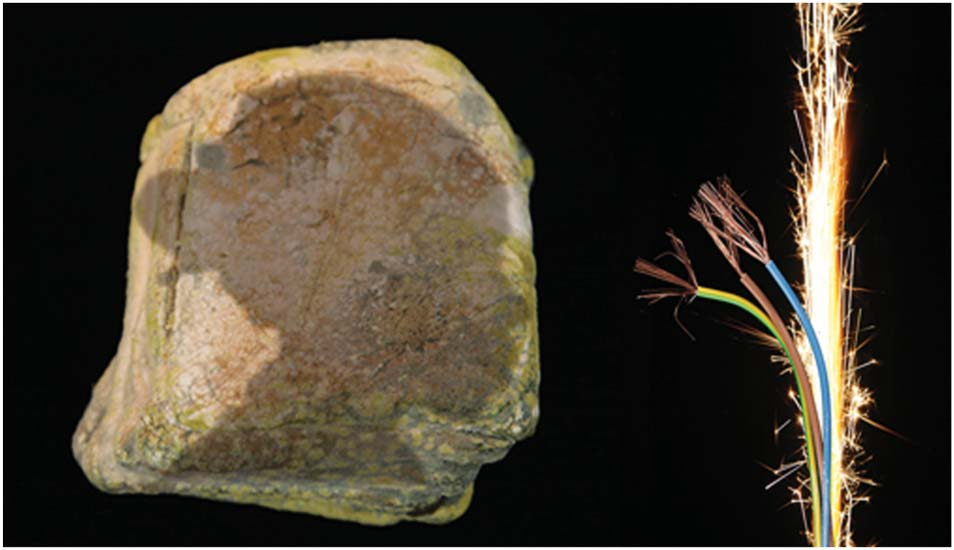
Left hand image: Deborah Padfield with Linda Williams, “Untitled” from the series Face2face, 2008–13. Digital Archival Prints. © Deborah Padfield. Right hand image: Deborah Padfield with Jillie Abbott, “Untitled” from the series Face2face, 2008–13. Digital Archival Prints. © Deborah Padfield.

An unexpected additional benefit of the projects was the therapeutic effect on those who participated in the cocreative image-making process. The artist, Padfield, was at pains from the outset to make clear that this was not art therapy. The aim was to cocreate strong images which could do justice to the intensity of patients' individual experiences and to communicate to a wider audience beyond the art room or any therapeutic dynamic. One ambition was to use these images to help reduce stigma and create a communication tool for use with future patients (Fig. 2).

The images and extracts of patient testimonies were exhibited in London hospitals and galleries including the Menier Gallery, Science Museum, and Wellcome, across the United Kingdom and internationally with supporting symposia and published as 2 books, *Perceptions of pain*,^64^ and along with wider analyses of the pain experience and the value of creative representations, *Encountering Pain*,^72^ as well as a series of academic essays.^4,67–69,73,86^

**Figure 3.**
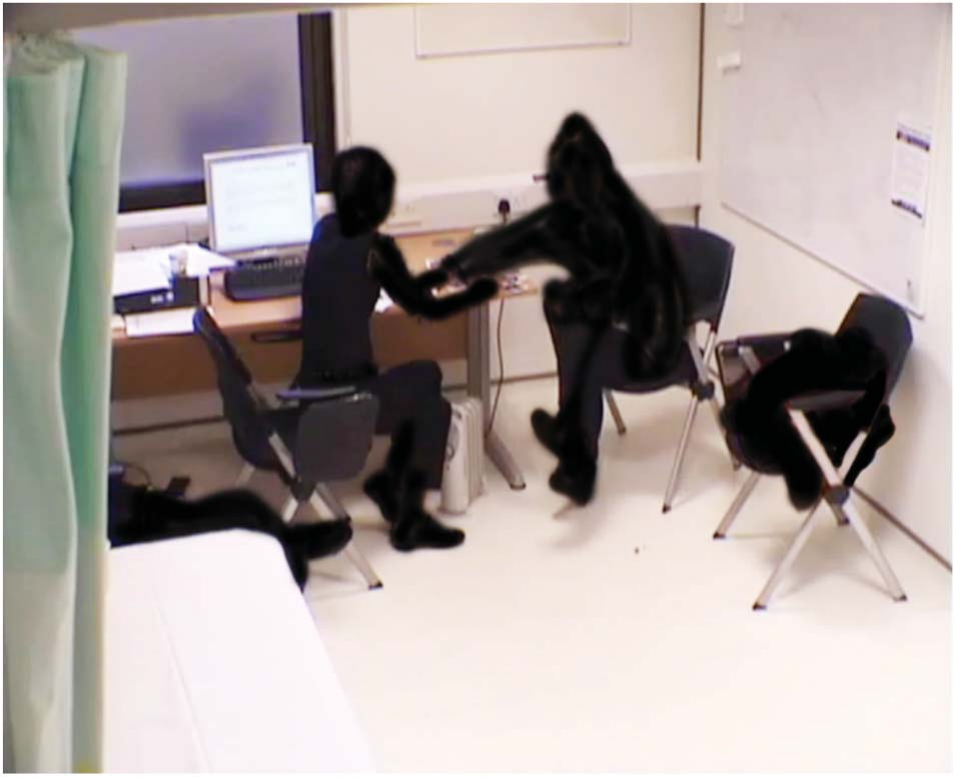
Anonymised screen grab of video footage from consultation with pain cards/images (filmed with consent) showing the active use of the space between the patient and the clinician.

A selection of the images were combined to create a pack of 54 laminated PAIN CARDS (images of pain approx. 6″ × 4″ like large playing cards) and were then offered to other patients not involved in creating them while they waited for their specialist pain consultation. They were given approximately 20 minutes to look through them and were invited to use them in any way they wished during the consultation to discuss their pain and its impact. These were routine NHS pain consultations where both parties need to obtain a history, diagnosis, and management plan. The consultations were video recorded and postconsultation questionnaires were completed independently by the patient and the clinician. These plus the filmed footage were subsequently analysed by a multidisciplinary team. In some ways, the team's multiple lenses could be seen as paralleling the multiple experiences of the same consultation for clinician, patient, and/or family.^69^ Through diverse methodologies, we found that the images encouraged discussion of the emotional components of pain, affecting both verbal and nonverbal aspects of the interaction; made the language more personal and increased affiliation behaviours, engaging both patient and doctor in more negotiated and democratised behaviour (Fig. 3).

## 6. Evaluation I: summary of findings of academic interdisciplinary research team

Details of findings from the academic multidisciplinary analyses are available,^4,17,62,65,67–69,72,86,101,103^ but are described here to demonstrate how the images gained agency themselves as well as conferring it on the patient. The language and behaviour in consultations compared patients who were offered the image cards with others who were not.

### 6.1. Linguistic analysis

Linguist, Semino observed that, in total word counts, patients speak more than clinicians during image use (card data), unlike the rest of the consultation.^84^ This difference in the volume of talk was found to be statistically significant according to the “log likelihood” test, which is commonly used in Corpus Linguistics (*P* < 0.0001). This speaks to ways in which the relative proportion of talk in interaction is a means through which “asymmetries of power in society generally and in institutional settings, in particular, can manifest themselves in interactions.”^93^ The increased amount of patient talk reverses the normal pattern seen in consultations and was accompanied by increased listening by clinicians. Semino also used the computer-aided programme Wmatrix to look at what kind of language is used around the cards vs when the cards are not being used. The types of words and areas of meaning that occurred statistically more frequently during image use^84^ included personal pronouns, perhaps indicating increased agency; maximisers (such as “absolutely,” “completely”); domains of thought and belief; and words and narratives reflecting emotional disclosure.^84^ The word “feel” was used to describe aspects of the pain experience: the quality of the pain sensation (I feel as if I am on fire); the impact of the pain on the person's daily life (eg,: “to feel that there are some things I am not able to do any more”); and the emotional consequences of chronic pain, also often involving figurative expressions (eg,: “I feel lost, I do not know what to do”).^84^ Semino noted how patients used the cards in particular to talk about their emotional and subjective states and the impact pain had on their lives. The cards effectively reduced power asymmetry between patient and clinician and provided greater opportunities for verbalisation of lived experiences of pain and in particular, emotional disclosure, which seemed to make it easier to address the totality of a person's needs and challenges within the therapeutic relationship.^84^

### 6.2. Nonverbal analysis

Nonverbal postures and movements indicating dominance, control, and affiliation^43,49^ were coded in clips drawn at regular times across the consultations^15^ and showed greater rapport and clinician–patient affiliation during image consultations.^73,86,101^ The images “gave patients the central role in explaining their pain experience, and most, if not all, used image cards to explore emotional dimensions as well as to elaborate on sensory descriptions”^101^ (p. 187). Although created with different patients from those who used them in this study, the images felt relevant for study patients with chronic pain, affecting behaviour of both patient and clinician, and facilitating emotional disclosure and rapport.^101^ As with language, use of the images made for a more equal relationship expressed nonverbally, with the clinician speaking less and listening more. “That listening in itself, with some open questions or encouragement to the patient to extend an initial explanation of what the image meant, may have been powerful in building and deepening rapport. It might not have occurred if the patient had, without cards, embarked on emotional disclosure”^101^ (p. 187).

Observing the footage, Padfield also found it interesting to note that when the images were not used, the physical space between clinician and patients was little used. With images, it was possible to observe a negotiated dance of limbs and cards into that space, a dynamic which seemed to continue into the rest of the consultation. This observation is supported by the nonverbal analysis, which also raised an additional finding we could not have anticipated, that the impact on clinicians' behaviour seemed greater than that on patients' behaviour, requiring further research to understand, but consistent with clinician report.^103^

### 6.3. Clinical response

In a chapter reflecting on her interest in the use of images in clinical spaces and in particular on the study described above, Zakrzewska^103^ picks up on the space between clinician and patient “… using images can change the dynamics … the PAIN CARDS … have made me appreciate the importance of the spaces between myself and the patient and how this can be effectively used for the sharing of information. The space enables material to be personalised and amplifies the patient's voice when making decisions”^103^ (p. 351).

Zakrzewska has changed her practice to actively use the space between herself and her patient, whether through drawing, use of the cards, sharing leaflets/drug regimens, or looking together at the computer screen.

### 6.4 “Art and agency”: observations from art psychotherapeutic theory

The art psychotherapist in our team drew on theories of art therapy normally applied to image-making^62^ to draw attention to the triangular relationship between patient, clinician, and image—referencing a theory of transactional objects where the images are handled and used by the client to have more control over aspects of the therapeutic relationship.^82,83^ This resonates with Gell's notion of the art object as relational^35^ and provides key insights into the way images work in social spaces such as the consulting room.

**Figure 4.**
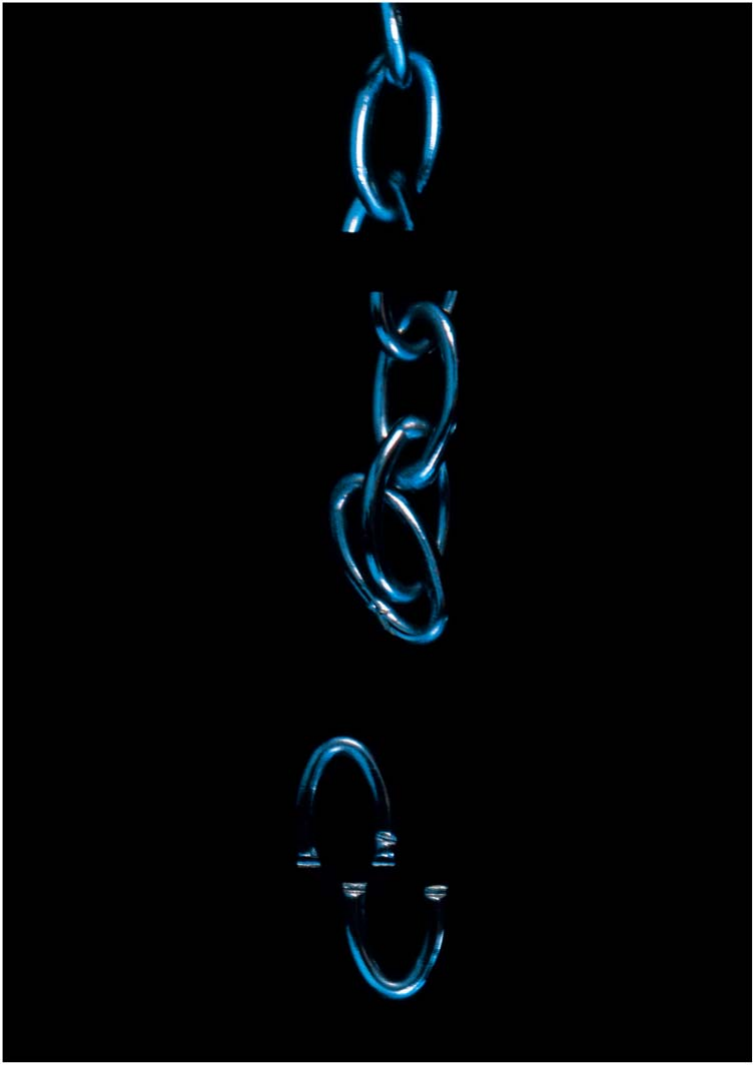
Deborah Padfield with John Pates, “Untitled” from the series Perceptions of Pain, 2001–06. Silver Gelatin Print. © Deborah Padfield, reproduced by kind permission of Dewi Lewis. NB, This image connoted a gap in the vertebrae for the original patient who cocreated it. For one of the patients viewing it in the clinic, it connoted a gap in the family.

**Figure 5.**
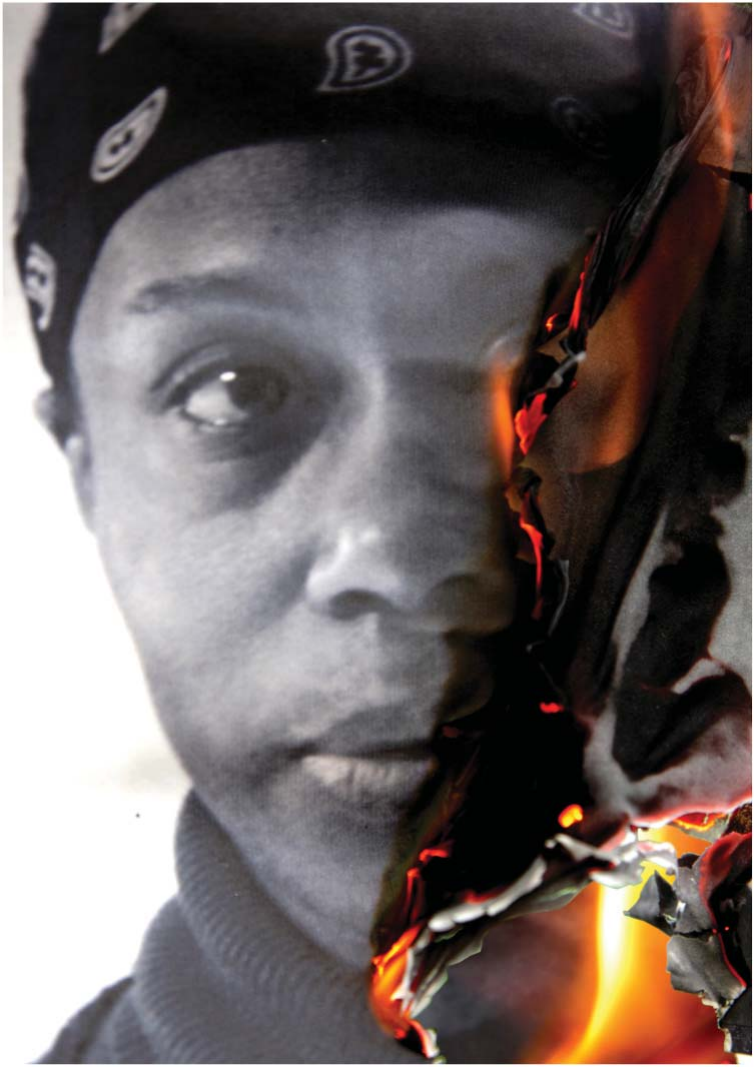
Deborah Padfield with Linda Williams, “Untitled” from the series Face2face, 2008–13. Digital Archival Prints. © Deborah Padfield. NB for the original patient who cocreated it the image connoted heat, for one of the patients viewing it in the clinic, it connoted a change in identity.

Agency could also be conceived through the images having been cocreated with other people living with pain. Something of their pain and body is present, through the images, in the consulting room, giving permission to describe pain in graphic ways such as: “*a huge piece of chewing gum … wrapped around my spine*”, “*Sometimes I feel a gap between my family …* they are not coming to visit for Christmas”, “*that person's face is burning off … my self-identity is being worn away*”, “*muffled like you have no voice, there is no mouth*”. At times, as patients lay the cards on the table, it seemed as though the cards conferred agency on the patient and provided a tool through which they led parts of the consultation. The process could be viewed as performances of identity construction and relationship building with the images, to use Gell's term, becoming “relational”^35^ (Figs. 4 and 5).

It is arguable that the images could also function as some sort of “transitional” object in a Winnicottian sense. Omand identifies how Winnicott's ideas about transitional objects and spaces can help us think about the image as occupying an “in-between” place.^69^ “They are a way of describing an in-between area where our subjective inner world experience meets the external world. The images are like transitional objects in that patients project their own subjective inner experiences onto something concrete and external (the image)”^69^ (p. 78). This imbues images with projected meaning and therefore contains “the stuff of the patient's inner world as well as existing materially, as perceived by both participants in common, to be looked at and thought about”^69^ (p. 78).

## 7. Evaluation II

The project was also reviewed by an independent evaluator of public engagement, who elicited in-depth first-hand experience of the process, the outcomes, and impact it had on 5 patients and their pain (Moore G, May 2016).

‘*The evaluation has* *identified* *how empowerment has taken place in those involved in the project, alongside the projects’ potential contribution to wider impacts on understandings and knowledge of facial pain*.’

### 7.1. Some of the patients' comments include

“I wanted to put my message across about what I had been through … and this project [face2face] was my way of doing that”

“it took me to thinking ahead, … to being more positive and thinking about more positive times. Somehow it got me out of the cycle I was in.”

“it was an excellent distraction from the pain.”

“I say to people I believe in the process of imagery and art as I used it for my own recovery. It did many, many things for me … and probably I would not be where I am now”.

### 7.2. Raising public awareness

Although the way in which the projects and allied public engagement activities such as exhibitions, workshops, films, symposia, and webinar, raised public awareness of pain and its challenges was not within the scope of her report, Moore highlighted:

‘*What these impacts illustrate is face2face is not simply a “project” or “set of activities” but a distinct model, offering an interrogation of experiences of facial pain in order to improve dialogues about pain. Through a range of activities, i.e. the co-creation of images, exhibitions, pain cards, face2face has embodied values of collaboration and participation in its approach and it is this approach which has encouraged learning, raised awareness of facial pain and improved personal well-being.*’

### 7.3. Patients' comments include

“I was in the newspaper as well… It was related to facial pain, and it was more to do with raising awareness of it.”

“Having this horrible illness has opened a door for me to create something to help other people to express their pain.”

“I think the pain cards will give people a sense, it will give a bigger picture about pain and trigeminal neuralgia, both to those that have never heard about trigeminal neuralgia and comfort to who are suffering.”

## 8. Concluding reflections and recommendations for the future

Images can make a consultation more patient-centred by eliciting the meaning of the pain for patients at that moment. They provide a means through which patients can lead the consultation and focus on what matters most to them. Images demand attentive listening on the part of the clinician, holding the space so that the clinician can park on one side the temptation to “do” and instead engage in a live act of “witnessing.”

Since the COVID-19 pandemic, there has been a significant increase in the use of telephone and video consultations. In this context, it is hard for clinicians to pick up clues from nonverbal behaviour, and equally hard for clinicians to show they are listening. Handing an image back and forth, giving control of this process to the patient, demonstrates the clinician is listening. It helps show the clinician is engaged in a collaborative process of dialogue and is willing to hear and believe the patient's story and interpretation. It initiates a negotiated interaction. In a world increasingly dependent on nonhuman interactions for diagnosis and care,^3^ it is more important than ever to use direct interaction to ensure patients feel listened to, that clinicians have heard what matters most to them and will include them in the choice of next steps.

**Figure 6.**
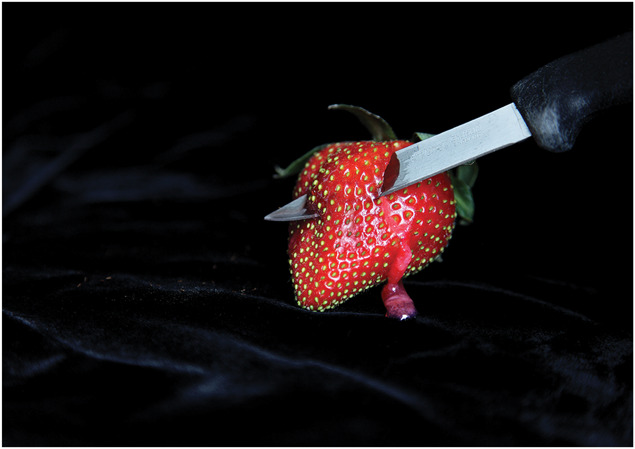
Deborah Padfield with Alison Glenn, “Untitled” from the series Face2face, 2008–13. Digital Archival Print. © Deborah Padfield. NB Figures 5 and 6 show an aspirational longitudinal series where in the first image, the strawberry with the knife through it connotes pain/injury and the second image connotes a sense of being able to throw away the icons of pain identity and suggests that the pain and not the self is the one now trapped behind an invisible barrier.

**Figure 7.**
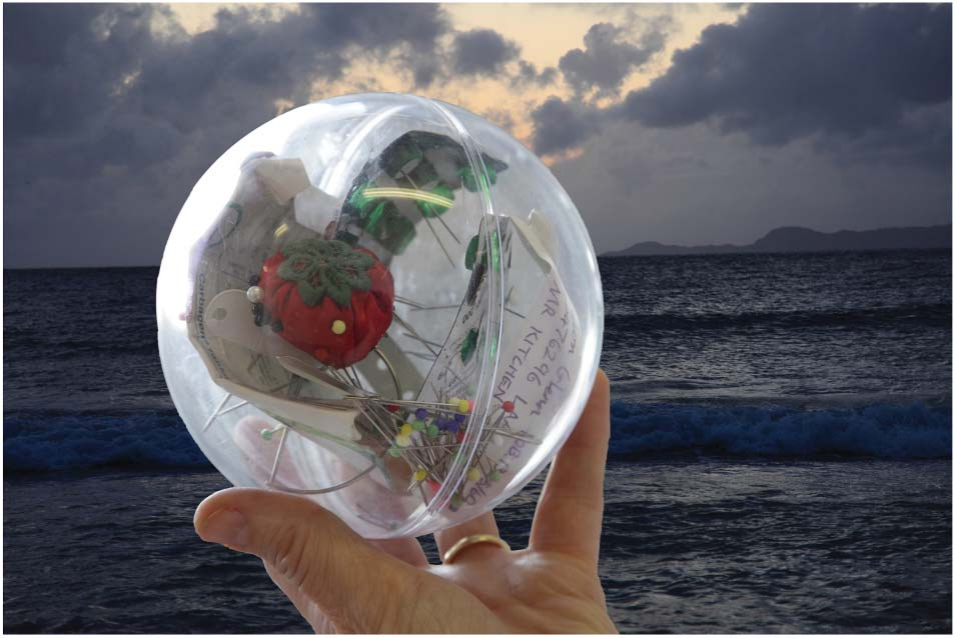
Deborah Padfield with Alison Glenn, “Untitled” from the series Face2face, 2008–13. Digital Archival Print. © Deborah Padfield.

In the physical shared space of face-to-face encounters, the images can also provide a reminder of the physicality of pain, shared reference points and engage all speakers in a process of negotiation. It offers people an opportunity to have an aspirational image in their head to lift them out of the present. Equally for the clinician, the images can support an MDT approach, visualising and mapping progress over time as patients' perception and understanding of their pain changes and the images reflect this; be used as metaphors to explain difficult concepts, and can make it easier for patients to explain their pain to family and friends (Figs. 6 and 7).

## 9. The future: transcultural work

**Figure 8.**
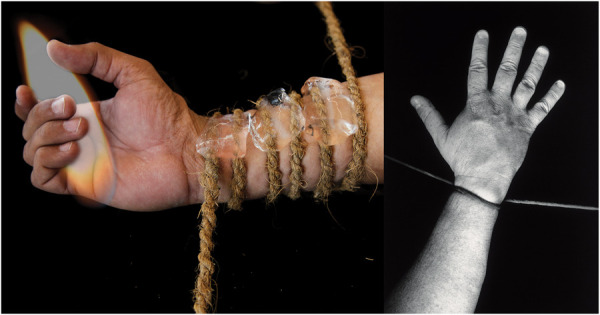
Left hand image made in Delhi, India: Deborah Padfield with Satendra Singh, “Untitled” from the series Visualising Pain, 2019 Digital Archival Print. © Deborah Padfield. Right hand image made in London, United Kingdom: Deborah Padfield with Robert Ziman-Bright, “Untitled” from the series Perceptions of Pain, 2001–06. Silver Gelatin Print. © Deborah Padfield, reproduced by kind permission of Dewi Lewis. The sense of being bound by an agent beyond the frame of the photograph seems to be a common if not universal theme in the images.

Literature reveals that ethnoculturally diverse patients ascribe pain and its meaning to diverse causes, affecting responses to treatment options/patient outcomes, and raising challenges for communication. Pain narratives are influenced by ways a person/community are connected to their sociocultural contexts and change over time,^11^ leading to pain meanings as diverse as fear and punishment or means of spiritual purification and growth. It is essential to understand how people visualise, perceive, and manage pain to improve pain management services and their equity. This requires truly international collaborative networks. Our exploration, initially in London, United Kingdom, and subsequently in pilot projects in Delhi, India,^71^ and Osaka, Japan, have generated a wide range of images and aesthetics. Many will argue that the images will always be culturally specific, and they would be right. However, the pilot images have also revealed some universal themes along with context-specific observations. Metaphors reoccurring within the images created in India, Japan, and the United Kingdom all include: the shadow, restriction, and weight; being bound by an agent beyond the frame of the photograph; being out of control; extreme temperature; a negative process such as rotting bread or fruit; tension; loss; and changes in identity. Is it possible that cocreating an international set of pain cards could provide an economic and fruitful starting point for pain conversations and assessment globally (Fig. 8)?

## 10. Recommendations

We recognise there is a need for caution when using images. They can elicit unexpected and intense emotions in patient and care giver. We would therefore always advocate having access to psychological support and/or onward referral available. When using images, we suggest clinicians leave time and space for the emotions uncovered to be explored before the patient leaves the consulting room. However, we believe it is preferable that these emotions are voiced and that leaving them unvoiced can affect negatively on pain intensity and prolongation. Dissecting them early in the consulting process can help build trust, reduce the need for unnecessary investigations, and save time (a precious commodity in most contexts) in the future.

We have therefore identified a range of ways in which this work could be built on, theoretically, clinically, and creatively (Table 1). It could also be expanded to other fields such as oncology, palliative care, bereavement, and disability studies.

**Table 1:** List of recommendations.

List of recommendations
Further theoretical research into the interaction of images and words within clinical encounters could help us better understand ways in which images can support clinician-patient interaction emotionally and cognitively
Larger scale quantitative studies of the impact of a bank of images on pain consultations
Use of images to open up topics of importance to patients, which they may have found difficult to raise
All undergraduate medical and healthcare courses should include a mandatory module in the arts or medical humanities
Use of images to support ‘attentive listening’ by clinicians and patients
Research into the use of images as a means of increasing clinician–patient mutual trust
Workshops for clinicians on using images and feeling confident to invite their patients to bring images to their consultations as springboards for discussion
Encourage patients to create their own photographic journey through their consultations and share with their healthcare providers (aware of powerful emotions these may raise)
Encourage active use of the space between clinician and patient
Use of images with patients who do not have a sufficient command of the native language
Further work on transcultural conceptions of pain and methods of management to learn from each other to find effective tools to support mutually beneficial communication and relieve the suffering and stigma so often associated with unresolved pain
Policy documents on pain should include input from artists, linguists, and patients with lived experience

There are many exciting developing projects using photography to picture health and reverse stories of trauma and stigma such as the new face of leprosy^44^ or to catalyse pain narratives, Mahdavi's *Flood of Dark*,^54^ a long-term project exploring how women with endometriosis conceptualize and communicate their pain—particularly through metaphor, narrative, and cocreated image-making and the *making the invisible visible* project—also on endometriosis pain.

We must not lose sight of the power of the arts to evoke emotion and call to collaborative action, to catalyse dialogue across perspectives and create and reveal new knowledge, understanding and hope. As artist, Grayson Perry, writes “Art, Like Science and Religion helps make meaning from our lives and to make meaning is to make us feel better.”^1^ Here art uncovers meaning, initiates more collaborative dialogue and thus “makes us feel better.” To quote Yawar in the Lancet, “… as well as being removed or shared, suffering *can* be transformed into knowledge, through being infused with meaning and purpose”.^102^ (p. 1285) (Table 2).

**Table 2:** Table of main empirical approaches.

Approaches and examples	Outcomes	Comments on rigor
Using photographic images cocreated by patients with pain and artist, used by those patients to convey meaning of pain in clinical settings.^64,65,70^	User experience, for example, patient satisfaction, clinician satisfaction.	Established qualitative and quantitative methods.
Using supplied photographic images (cocreated by other patients and artist) to improve clinical communication and interaction.^4,66,67,68,69,72,73,84,85,86,101^	User experience, for example, validation, satisfaction for patient, feelings of empathy for clinician. Language and nonverbal behaviour categorised for frequency and sequence.	Established qualitative and quantitative methods Potentially labour-intensive.
Pain drawing by patient to aid clinical assessment.^56,89,96^	Diagnostic accuracy using pain quality, quantity, location, representation.	Some judgements of aspects of drawing (eg, how pain is marked) highly subjective. Inconsistency and unreliability in diagnostic systems problematic.
Pain drawing by patient for pain expression.^48,60,76^	Patient experience for example, validation, satisfaction. Clinician experience for example, empathy.	Established qualitative and quantitative methods.
Association of arts involvement with health benefits including in relation to pain.^32,33^	Health, quality of life.	Naturalistic studies using robust epidemiological methods.
Arts interventions to benefit health, including social prescribing of arts.^1,2,22,30,55,78,92^	Quality of life, pain quality and quantity, pain interference, mood, function.	Draws on established quantitative and qualitative methodologies. Risks underpowered samples.
Art, film, or photographic workshops for those living with persistent pain and/or for healthcare professionals working in pain services.^65^	Pain expression, reduction of isolation, satisfaction in creative practice; feeling heard and understood.	Limited evaluation to date, for example, qualitative analysis of participant accounts and anecdotal evidence.
Training in use of images for healthcare professionals and students/trainees working with pain.^71,86^	Increased clinician confidence in using images in clinical setting; quality of communication with patients.	Limited evaluation to date, for example, qualitative analysis of participant accounts.

## Conflict of interest statement

The authors have no conflicts of interest to declare.
